# Developmental Regulation of the Murine Selenoproteome Across Embryonic and Postnatal Stages: Implications for Human Nutrition and Health

**DOI:** 10.3390/nu17203200

**Published:** 2025-10-11

**Authors:** Shan-Shan Wang, Tong Li, Cheng-Jia Wei, Lan-Yu Cui

**Affiliations:** 1 College of Food Science and Nutritional Engineering, China Agricultural University, Beijing 100083, China; 18064636815@163.com; 2 University Engineering Research Center of Advanced Technologies in Medical and Biological Intelligent Manufacturing, Guangxi Medical University, Key Laboratory of Longevity and Aging-Related Diseases of Chinese Ministry of Education, Institute of Neuroscience, School of Basic Medical Sciences, Guangxi Key Laboratory of Brain Science, Key Laboratory of Basic Research on Brain Function and Disease of Guangxi Health Commission, Nanning 530021, China; myqfchenguang@163.com; 3 Institute of Agricultural Products Preservation and Processing Technology (National Engineering and Technology Research Center for Preservation of Agricultural Products), Tianjin Academy of Agricultural Sciences, Key Laboratory of Storage and Preservation of Agricultural Products, Ministry of Agriculture and Rural Affairs, Tianjin 300384, China

**Keywords:** development, embryo, selenoprotein, mice, mRNA

## Abstract

**Background/Objectives:** Selenoproteins play indispensable roles in embryonic development, with their dysregulation linked to various metabolic and neurological disorders. This study aims to systematically quantify the mRNA expression levels of all 24 selenoprotein genes in murine heart, brain, liver, and kidney tissues across embryonic (E8.5, E12.5, E18.5) and postnatal (P7, P30, P90) developmental stages, in order to elucidate the regulatory landscape of selenium metabolism during development. **Methods:** We collected tissues from mice at six developmental stages and performed RNA extraction followed by quantitative real-time PCR (qPCR) to measure the expression of all 24 selenoprotein genes. Data were normalized using the geometric mean of ActB and Gapdh, and statistical analyses were conducted using one-way ANOVA with Duncan’s post hoc test. **Results:** Our analysis reveals three principal findings: (1) Distinct expression patterns emerge among selenoprotein families—deiodinases (*Dio*1-3) and thioredoxin reductases (*Txnrd*1-3) exhibit limited embryonic expression (<20-fold changes), while glutathione peroxidases (*Gpx*1, *Gpx*3, *Gpx*4) and biosynthesis-related genes (Selenop, *Msrb*1) show substantial postnatal upregulation (up to 600-fold increases); (2) Selenoproteins essential for embryonic survival (*Gpx*4, *Txnrd*1, *Txnrd*2, Selenoi, Selenot) display expression profiles concordant with their essential developmental functions; (3) Selenop and *Msrb*1, involved in selenium transport and redox regulation, demonstrate early embryonic upregulation with further increases during postnatal development. **Conclusions:** These spatiotemporal expression patterns elucidate the regulatory landscape of selenium metabolism during development and provide mechanistic insights into the phenotypes associated with selenium deficiency. The findings offer valuable implications for human nutritional interventions and developmental health.

## 1. Introduction

The biological functions of selenium (Se) are predominantly mediated through its incorporation into selenoproteins, which contain selenocysteine (Sec) as the 21st amino acid [1,2]. As an essential micronutrient, selenium must be acquired through diet, and its deficiency is associated with various human pathologies, including Keshan disease, cognitive decline, and impaired immune function. This incorporation requires a complex translational machinery that recognizes the UGA codon, typically a stop signal, to insert Sec. Alterations in selenoprotein gene expression have been implicated in various pathological conditions, including diabetes mellitus, cardiovascular diseases, and neurological disorders [3]. The human and rodent genomes encode 25 and 24 selenoprotein genes, respectively, representing a sophisticated system for maintaining redox homeostasis and regulating diverse metabolic pathways [4].

Functionally, selenoproteins can be classified into seven categories: (1) deiodinases (DIOs), which regulate thyroid hormone metabolism through reductive deiodination; (2) glutathione peroxidases (GPXs), responsible for detoxifying hydrogen peroxide and lipid hydroperoxides; (3) thioredoxin reductases (TXNRDs), which reduce thioredoxin and other substrates using NADPH; (4) other selenoenzymes including methionine sulfoxide reductase B1 (MSRB1) and selenoprotein I (SELENOI); (5) selenoprotein biosynthesis factors; (6) CXXU-motif-containing selenoproteins; and (7) endoplasmic reticulum (ER)-resident selenoproteins.

The DIO family comprises three isoforms (*Dio*1-3) that critically regulate thyroid hormone activity in a tissue-specific manner. Thyroid hormones are essential for human growth, development, and metabolism, and selenium availability directly influences their activation. The GPX family performs diverse functions in H_2_O_2_ signaling, lipid peroxide detoxification, and maintenance of cellular redox homeostasis [5], exhibiting distinct tissue-specific expression patterns (e.g., *Gpx*2 predominantly in gastrointestinal tract and liver, *Gpx*3 in kidney, and *Gpx*4 highly expressed in testis with ubiquitous distribution). TXNRDs facilitate thioredoxin reduction using NADPH, participating not only in antioxidant defense but also in regulation of transcription factors and apoptotic pathways. Although *SELENOP* expression occurs in multiple tissues [6], hepatic SELENOP serves as the primary selenium transport protein to extrahepatic tissues. This transport function is crucial for distributing selenium to tissues such as the brain and endocrine organs, which is particularly relevant for human health. SEPHS2 catalyzes monoselenophosphate formation from selenide and ATP, essential for selenocysteine incorporation into all selenoproteins [7]. MSRB1 specifically reduces methionine-R-sulfoxides in proteins, playing crucial roles in protein repair mechanisms, while SELENOI catalyzes phosphoethanolamine transfer from CDP-ethanolamine to diacylglycerol to form phosphatidylethanolamine, involved in phospholipid metabolism.

The CXXU-motif-selenoproteins (including SELENOF, H, M, O, T, V, and W) may possess oxidoreductase activity, though their precise physiological functions remain largely enigmatic. The ER-resident selenoproteins (SELENOK, N, and S) participate in oxidative stress response, calcium flux regulation, and ER-associated degradation, maintaining ER homeostasis [8]. Dysregulation of ER homeostasis is implicated in human neurodegenerative diseases, suggesting a potential link between selenoprotein function and neurological health.

The fundamental essentiality of the entire selenoproteome is demonstrated by the embryonic lethality observed in Sec-tRNA[Ser]Sec knockout mice [9]. Among the 24 rodent selenoproteins, five have been established as individually essential for embryonic survival: Gpx4^−^/^−^ (E7.5) [10], Txnrd1^−^/^−^ (E8.5–10.5) [11], Txnrd2^−^/^−^ (E13.5) [12], Selenoi^−^/^−^ (E16.5–18.5) [13], and Selenot^−^/^−^ (E8) [14]. In contrast, knockouts of other selenoprotein genes generally remain viable, though often accompanied by metabolic alterations and increased stress susceptibility in adulthood [13,15,16,17,18,19,20]. This dichotomy raises the compelling question of whether essential and non-essential selenoproteins exhibit distinct spatiotemporal expression patterns during critical periods of embryonic development, neonatal life, and adolescence, potentially reflecting their differential functional importance—a question with direct relevance to human developmental biology and nutritional science.

Murine embryogenesis is conventionally divided into early (E8.5), middle (E12.5), and late (E18.5) stages, each characterized by specific developmental milestones [21]. Cardiac development initiates first, followed sequentially by brain, liver, and kidney formation [22]. At E8.5, the heart becomes morphologically identifiable as a linear tube within the pericardial cavity, while other organs remain incompletely formed, necessitating whole-embryo sampling for analysis. By E12.5, the heart, liver, kidney, and brain become readily discernible under microscopic examination. At E18.5, organs approach functional maturity and the fetus achieves viability ex utero [20,23]. Postnatal development encompasses the neonatal period (first 7 days, P7), a phase of rapid growth and development (P30), and sexual maturity (P90).

Despite the recognized importance of selenium and selenoproteins in development and metabolism, a comprehensive understanding of selenoprotein developmental regulation across multiple organs remains limited, particularly in the context of human nutritional requirements. Previous investigations typically focused on individual selenoproteins or restricted developmental windows, leaving significant gaps in knowledge regarding global selenoproteome dynamics during development. This study aims to address these gaps through systematic quantification of mRNA levels for all 24 selenoprotein genes in four vital organs (heart, brain, liver, and kidney) across six critical developmental stages. Our objectives include: (1) establishing a complete developmental atlas of the murine selenoproteome; (2) identifying differential expression patterns between essential and non-essential selenoproteins; (3) elucidating tissue-specific regulatory mechanisms; and (4) providing insights into the functional implications of selenoprotein expression dynamics for embryonic survival and postnatal metabolic maturation, with potential applications for optimizing human maternal and early-life nutrition.

## 2. Materials and Methods

### 2.1. Animals and Experimental Design

All experimental procedures involving animals were reviewed and approved by the Laboratory Animal Welfare and Animal Experimentation Ethics Committee of China Agricultural University (Approval Code: AW51905202-5-01) and were conducted in strict compliance with the Guidelines for the Care and Use of Laboratory Animals issued by the Ministry of Science and Technology of the People’s Republic of China. Eight-week-old Balb/c mice were obtained from Vital River Laboratory Animal Technology Co., Ltd. (Beijing, China), a licensed vendor operating under specific pathogen-free (SPF) conditions. Animals were housed under standard laboratory conditions with ambient temperature maintained at 22–25 °C, relative humidity at 40–60%, and a 12 h light/dark cycle (lights on at 08:00 h). Mice had ad libitum access to autoclaved drinking water and a standard pelleted diet (AIN-93G formulation, containing 0.15 mg Se/kg as sodium selenite). This selenium concentration is nutritionally adequate and comparable to recommended human dietary selenium intake levels, making our findings relevant for translational nutrition research. Dietary selenium content was verified by atomic absorption spectroscopy to ensure nutritional adequacy.

Following a 7-day acclimation period, sexually mature females were paired with males overnight. The presence of a vaginal plug on the following morning was designated as 0.5 days post-conception (dpc). Pregnant females were subsequently individually housed and monitored daily for health status and body weight changes.

### 2.2. Tissue Collection and Processing

Embryos were collected at three designated embryonic time points: E8.5, E12.5, and E18.5 (*n* = 6 per group). At E8.5, whole embryos were collected without micro-dissection due to their minute size and ongoing organogenesis. For E12.5 and E18.5 embryos, heart, liver, kidney, and brain tissues were meticulously dissected under a stereomicroscope (Leica M80, Germany) using micro-dissection instruments. Postnatal samples were collected from male mice at P7, P30, and P90 (*n* = 6 per group). All dissections were performed between 09:00 and 11:00 h to minimize potential confounding effects of circadian rhythm.

Immediately following collection, tissues were rinsed in ice-cold RNase-free phosphate-buffered saline (PBS, pH 7.4) to remove blood and contaminants, then minced into small fragments (approximately 2–3 mm^3^) using sterile surgical scissors. Tissue aliquots were snap-frozen in liquid nitrogen and stored at −80 °C until RNA extraction. All procedures from tissue collection to freezing were completed within 5 min to preserve RNA integrity.

### 2.3. RNA Extraction and Quality Assessment

Total RNA was isolated from approximately 30–50 mg of tissue using TRIzol reagent (Invitrogen, Carlsbad, CA, USA) according to the manufacturer’s instructions. Briefly, tissues were homogenized in 1 mL TRIzol using a Precellys Evolution homogenizer (Bertin Technologies, Montigny-le-Bretonneux, FranceFrance) with ceramic beads (1.4 mm diameter) at 6500 rpm for 20 s. Following complete dissociation of nucleoprotein complexes, chloroform was added (0.2 mL per 1 mL TRIzol). Samples were vigorously shaken for 15 s and incubated at room temperature for 3 min prior to centrifugation at 12,000× *g* for 15 min at 4 °C. The aqueous phase was transferred to a new tube, and RNA was precipitated with isopropyl alcohol (0.5 mL per 1 mL TRIzol). The RNA pellet was washed twice with 75% ethanol, air-dried for 5 min, and resuspended in RNase-free water.

RNA concentration and purity were determined using a NanoDrop Lite spectrophotometer (Thermo Scientific, Wilmington, DE, USA). Samples with A260/A280 ratios between 1.8 and 2.1 and A260/A230 ratios greater than 2.0 were deemed acceptable. RNA integrity was further assessed by 1.5% agarose gel electrophoresis, which confirmed clear 28S and 18S ribosomal RNA bands without significant degradation. Only RNA samples with RNA integrity number (RIN) values > 7.0, as determined by an Agilent 2100 Bioanalyzer (Agilent Technologies, Santa Clara, CA, USA), were utilized for subsequent analysis.

### 2.4. cDNA Synthesis

Complementary DNA (cDNA) was synthesized from 1 μg of total RNA using the PrimeScript™ II 1st Strand cDNA Synthesis Kit (TaKaRa, Kyoto, Japan) in accordance with the manufacturer’s protocol. The reaction mixture contained 1× PrimeScript II Buffer, 0.5 mM dNTP Mixture, 0.5 μM Oligo dT Primer, 0.5 μM Random 6 mers, 40 U RNase Inhibitor, and 100 U PrimeScript II RTase in a total volume of 20 μL. The reverse transcription reaction was carried out at 42 °C for 45 min, followed by enzyme inactivation at 95 °C for 5 min. The resultant cDNA was diluted 1:5 with nuclease-free water and stored at −20 °C until further use.

### 2.5. Quantitative Real-Time PCR (qPCR) Analysis

Gene-specific primers for all 24 selenoprotein genes and two reference genes (ActB and Gapdh) were designed using Primer Express 3.0 software (Applied Biosystems, Foster City, CA, USA) and synthesized by Sangon Biotech (Shanghai, China). All primer sequences are provided in Table 1. Primer specificity was validated by melt curve analysis and agarose gel electrophoresis of PCR products. Primer sequences were designed to amplify all known splice variants of each selenoprotein gene where possible, based on current annotations in the NCBI RefSeq database. However, due to the potential existence of unknown splice variants and the focus on total mRNA expression, the primers may not detect all variants.

Quantitative real-time PCR (qPCR) was performed using a CFX96 Touch Real-Time PCR Detection System (Bio-Rad, Hercules, CA, USA) with SYBR Premix Ex Taq II (Tli RNaseH Plus, TaKaRa). Each 20 μL reaction contained 10 μL of 2× SYBR Premix Ex Taq II, 0.4 μM of each forward and reverse primer, 2 μL of diluted cDNA template, and nuclease-free water. The thermal cycling protocol consisted of an initial denaturation at 95 °C for 1 min, followed by 40 cycles of 95 °C for 10 s, 58 °C for 10 s, and 72 °C for 10 s. A melt curve analysis was performed from 65 °C to 95 °C with increments of 0.5 °C every 5 s to verify amplification specificity. All samples were analyzed in triplicate, and no-template controls were included in each run to monitor for potential contamination. Ct values > 35 cycles in all replicates were considered non-detectable (not expressed).

### 2.6. Data Analysis and Normalization

Relative mRNA expression levels were calculated using the comparative Ct (2^−ΔΔCt^) method [23]. The geometric mean of the Ct values of ActB and Gapdh was used for normalization, as these reference genes were validated to exhibit stable expression across all developmental stages and tissues as determined by geNorm and NormFinder algorithms [24,25,26]. The expression level at E8.5 (for embryonic comparisons) was set as the calibrator (1.0-fold). PCR efficiency for each primer pair, determined using serial dilutions of cDNA, ranged from 90% to 110%, with correlation coefficients (R^2^) exceeding 0.99.

### 2.7. Statistical Analysis

Data are presented as mean ± standard error of the mean (SEM) of six biological replicates. Statistical analyses were performed using SPSS version 13.0 (SPSS Inc., Chicago, IL, USA). For each selenoprotein gene within each tissue, one-way analysis of variance (ANOVA) was employed to assess the effect of developmental stage on mRNA expression. When ANOVA indicated significant differences (*p* < 0.05), Duncan’s multiple range test was applied for post hoc comparisons between specific time points. A probability value of *p* < 0.05 was considered statistically significant. Data visualization was performed using GraphPad Prism version 8.0 (GraphPad Software, San Diego, CA, USA).

## 3. Results

### 3.1. Expression Patterns of Selenoprotein Genes in the Heart

**Figure 1:**
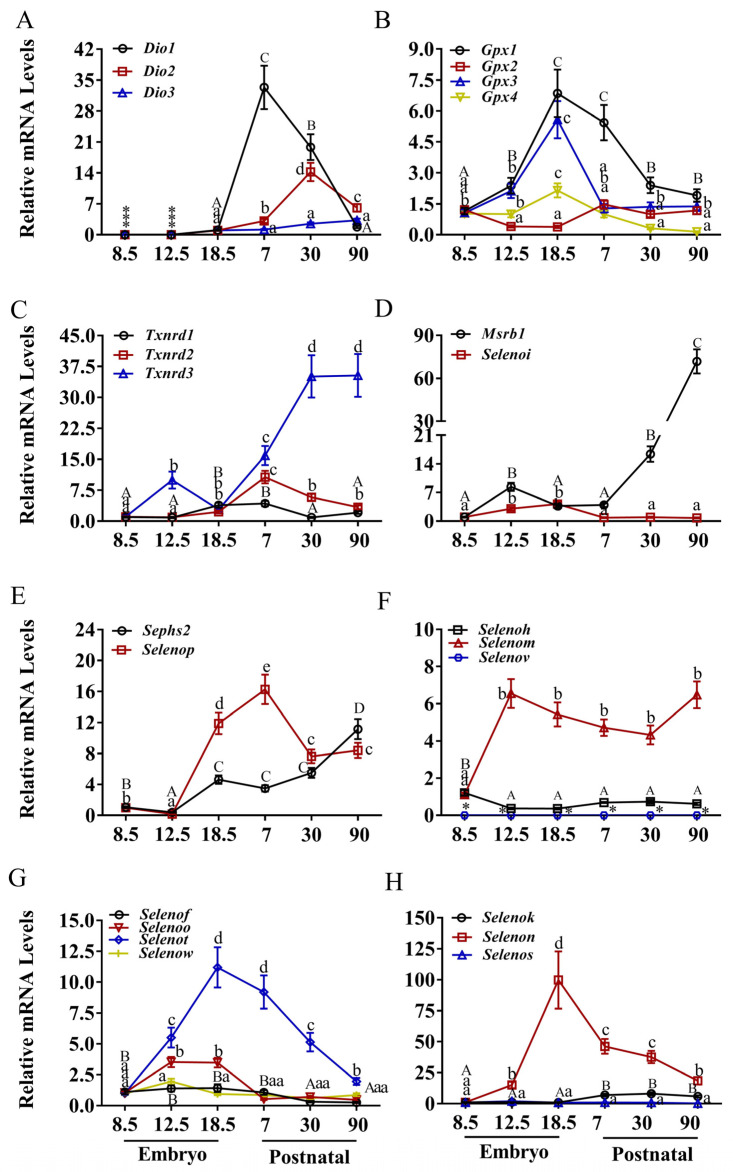
Expression of selenoprotein genes in heart of mice. (**A**) The deiodinase family, (**B**) the glutathione peroxidase, (**C**) the thioredoxin reductase family, (**D**) MRSB1 and SELENOI, (**E**) SEPHS2 and SELENOP, (**F**) CXXU-motif-containing selenoproteins (H, M, V), (**G**) CXXU-motif-containing selenoproteins (F, O, T, W), (**H**) ER-resident selenoproteins (K, N, S). * indicates that mRNA is not expressed. Values are means ± SEM (*n* = 6). Means that do not share a common superscript letter are different (*p* < 0.05) by Duncan’s multiple range test. The letters are assigned independently for each gene within a panel. Abbreviations: CXXU, Cys-x-x-Sec; *Dio*1, Iodothyronine deiodinases 1; *Dio*2, Iodothyronine deiodinases 2; *Dio*3, Iodothyronine deiodinases 3; *Gpx*1, glutathione peroxidase 1; *Gpx*2, glutathione peroxidase 2; *Gpx*3, glutathione peroxidase 3; *Gpx*4, glutathione peroxidase 4; *Msrb*1, Selenoprotein R; *Selenof*, Selenoprotein F; *Selenoh*, Selenoprotein H; *Selenoi*, Selenoprotein I; *Selenok*, Selenoprotein K; *Selenom*, Selenoprotein M; *Selenon*, Selenoprotein N; *Selenoo*, Selenoprotein O; *Selenop*, Selenoprotein P; *Selenot*, Selenoprotein T; *Selenov*, Selenoprotein V; *Selenow*, Selenoprotein W; *Sephs2*, Selenophosphate synthetase 2; *Txnrd*1, Thioredoxin reductase 1; *Txnrd*2, Thioredoxin reductase 2; T*xnrd*3, Thioredoxin reductase 3.

The heart exhibited distinct temporal expression profiles for various selenoprotein genes throughout development (Figure 1 and Figure S1). Transcripts of the deiodinase family genes (*Dio*1–3) were nearly undetectable during embryogenesis (Figure 1A). Postnatally, *Dio*1 expression increased markedly (~35-fold, *p* < 0.05) by P7, then declined sharply to baseline levels by P90, indicating tight and transient regulation after birth. *Dio*2 demonstrated its most pronounced increase between P7 and P30 (14-fold, *p* < 0.05), whereas *Dio*3 expression remained consistently low during the postnatal period. Notably, both *Dio*1 and *Dio*2 returned to near-baseline expression levels by P90.

Among glutathione peroxidases (Figure 1B), *Gpx*2 and *Gpx*4 mRNA levels remained stable at low levels across all developmental stages. In contrast, *Gpx*1 and *Gpx*3 increased sharply (4–5-fold, *p* < 0.05) between E8.5 and E18.5. After peaking at E18.5, *Gpx*3 dropped to baseline levels by P7, while *Gpx*1 expression, although reduced from its peak, remained elevated at P30 and P90 compared to E8.5 levels.

Thioredoxin reductase gene expression analysis (Figure 1C) revealed that Txnrd1 and Txnrd2 were expressed at low and relatively stable levels across most stages, with modest elevations observed between E18.5 and P7. Txnrd3 increased moderately between E8.5 and E12.5, and substantially (>30-fold, *p* < 0.05) between E18.5 and P30, maintaining elevated expression through P90.

*Msrb*1 expression increased gradually from E8.5 to P7, then rose sharply (>80-fold, *p* < 0.05) from P7 to P90 (Figure 1D). *Selenoi* expression increased moderately from E8.5 to E12.5 but declined to minimal levels from P7 to P90. *Sephs*2 expression rose steadily from E12.5 to P90 (Figure 1E), while *Selenop* increased 15-fold from E12.5 to P7 before decreasing by approximately 50% by P30.

Among CXXU-motif-containing selenoproteins (Figure 1F), *Selenoh* increased from E8.5 and plateaued after E12.5. *Selenom* expression rose initially from E8.5 to E12.5 and was maintained at moderate levels throughout development. *Selenov* mRNA was undetectable in cardiac tissue at all stages examined. As shown in Figure 1G, *Selenot* exhibited the most dynamic expression profile among CXXU-motif-selenoproteins, increasing linearly from E8.5 to E18.5 before decreasing progressively through P90. *Selenoo* showed the second most variable expression pattern, while *Selenof* and *Selenow* were expressed at consistently low levels. *Selenok* expression demonstrated an upward trend over time, albeit at very low levels (Figure 1H). *Selenon* increased approximately 100-fold from E8.5 to E18.5, then decreased by approximately 50% at P7 and continued to decline through P90. *Selenos* expression remained low and stable during postnatal development.

### 3.2. Expression Patterns of Selenoprotein Genes in the Brain

**Figure 2:**
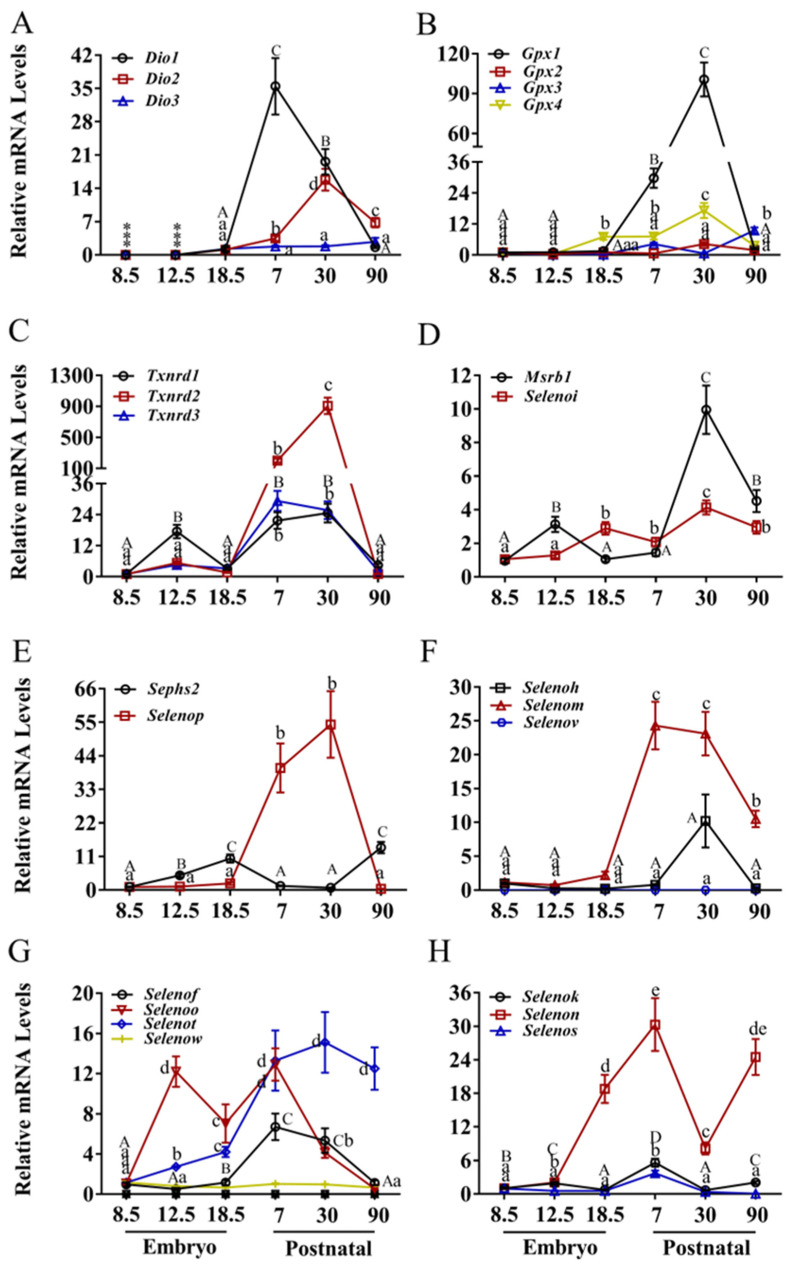
Expression of selenoprotein genes in brain of mice. (**A**) The deiodinase family, (**B**) the glutathione peroxidase, (**C**) the thioredoxin reductase family, (**D**) MRSB1 and SELENOI, (**E**) SEPHS2 and SELENOP, (**F**) CXXU-motif-containing selenoproteins (H, M, V), (**G**) CXXU-motif-containing selenoproteins (F, O, T, W), (**H**) ER-resident selenoproteins (K, N, S). * indicates that mRNA is not expressed. Values are means ± SEM (*n* = 6). Means that do not share a common superscript letter are different (*p* < 0.05) by Duncan’s multiple range test. The letters are assigned independently for each gene within a panel. Abbreviations: CXXU, Cys-x-x-Sec; *Dio*1, Iodothyronine deiodinases 1; *Dio*2, Iodothyronine deiodinases 2; *Dio*3, Iodothyronine deiodinases 3; *Gpx*1, glutathione peroxidase 1; *Gpx*2, glutathione peroxidase 2; *Gpx*3, glutathione peroxidase 3; *Gpx*4, glutathione peroxidase 4; *Msrb*1, Selenoprotein R; *Selenof*, Selenoprotein F; S*elenoh*, Selenoprotein H; *Selenoi*, Selenoprotein I; *Selenok*, Selenoprotein K; *Selenom*, Selenoprotein M; *Selenon*, Selenoprotein N; *Selenoo*, Selenoprotein O; *Selenop*, Selenoprotein P; *Selenos*, Selenoprotein S; *Selenot*, Selenoprotein T; *Selenov*, Selenoprotein V; *Selenow*, Selenoprotein W; *Sephs2*, Selenophosphate synthetase 2; *Txnrd*1, Thioredoxin reductase 1; *Txnrd*2, Thioredoxin reductase 2; *Txnrd*3, Thioredoxin reductase 3.

The brain displayed unique expression profiles distinct from other tissues (Figure 2 and Figure S2). The expression patterns of *Dio*1, *Dio*2, and *Dio*3 in the brain (Figure 2A) closely resembled those observed in the heart, with minimal expression during embryogenesis and postnatal induction. *Gpx*2 and *Gpx*3 expression remained low and stable across all stages (Figure 2B), while *Gpx*1 increased sharply (>90-fold, *p* < 0.05) from E18.5 to P30 before decreasing rapidly to baseline by P90. *Gpx*4 expression increased from E12.5, peaked at P30, and returned to baseline by P90.

*Txnrd*1, *Txnrd*2, and *Txnrd*3 expression (Figure 2C) was generally elevated postnatally compared to embryogenesis. *Txnrd*1 peaked at E12.5 and again at P30, while *Txnrd*2 rose sharply to a pronounced peak at P30. *Msrb*1 increased slightly between E8.5 and E12.5 and more substantially between P7 and P30, then declined from both peaks (Figure 2D). *Selenoi* increased steadily from E12.5 to P30. *Sephs*2 expression increased from E8.5 to E18.5, decreased until P30, and rose again at P90 (Figure 2E). *Selenop* was low during embryogenesis, increased 50-fold between E18.5 and P30, and returned to baseline by P90.

Among CXXU-motif-selenoproteins (Figure 2F), *selenom* was low during embryogenesis, increased sharply (>20-fold, *p* < 0.05) between P7 and P30, and then decreased by P90. *Selenoh* peaked at P30, increasing >10-fold from E18.5 before decreasing sharply. *Selenov* was undetectable in brain tissue. As shown in Figure 2G, *Selenot* increased from E8.5 to P7 and remained moderately elevated thereafter. *Selenof* increased from E8.5, peaked at P7, and declined to baseline by P90. *Selenoo* increased >12-fold from E8.5 to a peak at E12.5, decreased 5-fold by E18.5, peaked again at P7, and then decreased linearly to baseline. *Selenow* remained low and stable (Figure 2H). *Selenok* and *Selenos* were stable or low, with moderate increases between E18.5 and P7. *Selenon* increased >25-fold from E12.5 to a peak at P7, decreased 25-fold by P30, and peaked again at P90.

### 3.3. Expression Patterns of Selenoprotein Genes in the Liver

**Figure 3:**
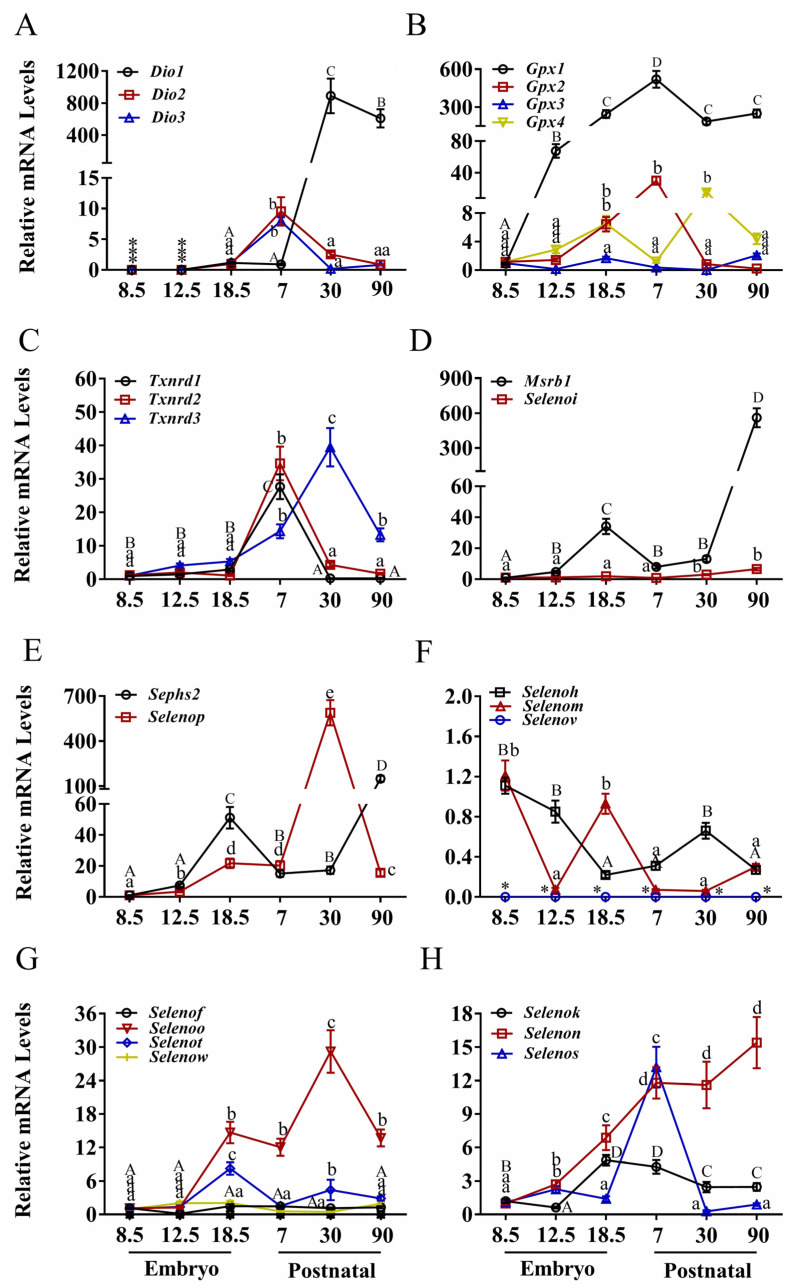
Expression of selenoprotein genes in liver of mice. (**A**) The deiodinase family, (**B**) the glutathione peroxidase, (**C**) the thioredoxin reductase family, (**D**) MRSB1 and SELENOI, (**E**) SEPHS2 and SELENOP, (**F**) CXXU-motif-containing selenoproteins (H, M, V), (**G**) CXXU-motif-containing selenoproteins (F, O, T, W), (**H**) ER-resident selenoproteins (K, N, S). * indicates that mRNA is not expressed. Values are means ± SEM (*n* = 6). Means that do not share a common superscript letter are different (*p* < 0.05) by Duncan’s multiple range test. The letters are assigned independently for each gene within a panel. Abbreviations: CXXU, Cys-x-x-Sec; *Dio*1, Iodothyronine deiodinases 1; *Dio*2, Iodothyronine deiodinases 2; *Dio*3, Iodothyronine deiodinases 3; *Gpx*1, glutathione peroxidase 1; *Gpx*2, glutathione peroxidase 2; *Gpx*3, glutathione peroxidase 3; *Gpx*4, glutathione peroxidase 4; *Msrb*1, Selenoprotein R; *Selenof*, Selenoprotein F; *Selenoh*, Selenoprotein H; *Selenoi*, Selenoprotein I; *Selenok*, Selenoprotein K; *Selenom*, Selenoprotein M; *Selenon*, Selenoprotein N; *Selenoo*, Selenoprotein O; *Selenop*, Selenoprotein P; *Selenos*, Selenoprotein S; *Selenot*, Selenoprotein T; S*elenov*, Selenoprotein V; *Selenow*, Selenoprotein W; *Sephs*2, Selenophosphate synthetase 2; *Txnrd*1, Thioredoxin reductase 1; *Txnrd*2, Thioredoxin reductase 2; *Txnrd*3, Thioredoxin reductase 3.

The liver, being a central organ in selenium metabolism, exhibited distinctive and dynamic expression patterns for various selenoprotein genes (Figure 3 and Figure S3). Consistent with observations in other tissues, transcripts of *Dio* genes were nearly undetectable during embryogenesis (Figure 3A). Postnatally, *Dio*1 expression increased dramatically (>800-fold, *p* < 0.05) from P7 to P30, followed by a substantial decrease (approximately 200-fold) by P90. *Dio*2 and *Dio*3 exhibited moderate increases (>5-fold) from E18.5 to P7 before declining to baseline levels.

*Gpx*3 remained stable at low levels across developmental stages (Figure 3B), whereas *Gpx*1 increased markedly (>500-fold, *p* < 0.05) from E8.5 to P7, then decreased approximately 200-fold by P90. *Gpx*2 increased substantially (>25-fold) from E12.5 to P7 before declining, while *Gpx*4 displayed an early peak at E18.5 and a later peak at P30.

Expression of *Txnrd*1, *Txnrd*2, and *Txnrd*3 (Figure 3C) was significantly higher during postnatal stages compared to embryogenesis. *Txnrd*1 and *Txnrd*2 increased sharply (~30-fold, *p* < 0.05) from E18.5 to P7 before decreasing rapidly. *Txnrd*3 increased steadily from E18.5, reached its peak at P30, and subsequently declined. *Msrb*1 exhibited an early peak at E18.5 (~30-fold) and a later, more pronounced peak at P90 (~500-fold) (Figure 3D). *Selenoi* maintained low and stable expression levels throughout development. *Sephs*2 peaked at E18.5 (~50-fold) and again at P90 (>100-fold) (Figure 3E). *Selenop* increased steadily from E12.5 to P7, peaked at P30 (~500-fold), and then decreased sharply.

*Selenoh* decreased progressively from E8.5 to P7 and remained at low levels thereafter (Figure 3F). *Selenom* decreased to low levels from E12.5 through P90. *Selenov* mRNA was undetectable in hepatic tissue. *Selenof* and *Selenow* exhibited stable but low expression levels (Figure 3G). *Selenoo* increased approximately 15-fold from E12.5 to P7, continued to rise until P30, and then decreased approximately 15-fold by P90. *Selenot* increased more than 8-fold from E18.5 before declining to baseline levels. Among ER-resident selenoproteins (Figure 3H), *Selenok* increased moderately (~5-fold) from E12.5 to E18.5 and remained stable. *Selenon* increased substantially (~15-fold) across developmental stages. *Selenos* increased more than 12-fold from E18.5 to P7 before decreasing to baseline levels.

### 3.4. Expression Patterns of Selenoprotein Genes in the Kidney

**Figure 4:**
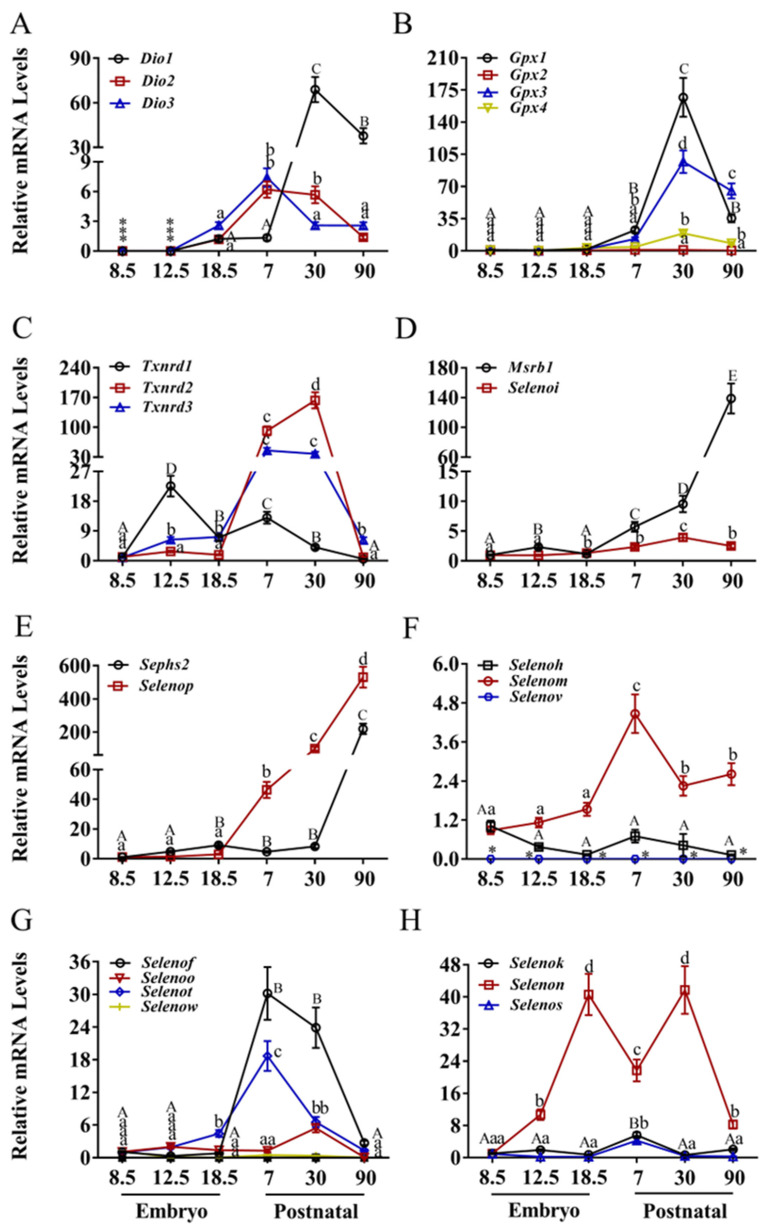
Expression of selenoprotein genes in kidney of mice. (**A**) The deiodinase family, (**B**) the glutathione peroxidase, (**C**) the thioredoxin reductase family, (**D**) MRSB1 and SELENOI, (**E**) SEPHS2 and SELENOP, (**F**) CXXU-motif-containing selenoproteins (H, M, V), (**G**) CXXU-motif-containing selenoproteins (F, O, T, W), (**H**) ER-resident selenoproteins (K, N, S). * indicates that mRNA is not expressed. Values are means ± SEM (*n* = 6). Means that do not share a common superscript letter are different (*p* < 0.05) by Duncan’s multiple range test. The letters are assigned independently for each gene within a panel. Abbreviations: CXXU, Cys-x-x-Sec; *Dio*1, Iodothyronine deiodinases 1; *Dio*2, Iodothyronine deiodinases 2; *Dio*3, Iodothyronine deiodinases 3; *Gpx*1, glutathione peroxidase 1; *Gpx*2, glutathione peroxidase 2; *Gpx*3, glutathione peroxidase 3; *Gpx*4, glutathione peroxidase 4; *Msrb*1, Selenoprotein R; *Selenof*, Selenoprotein F; *Selenoh*, Selenoprotein H; *Selenoi*, Selenoprotein I; *Selenok*, Selenoprotein K; *Selenom*, Selenoprotein M; *Selenon*, Selenoprotein N; *Selenoo*, Selenoprotein O; *Selenop*, Selenoprotein P; *Selenos*, Selenoprotein S; *Selenot*, Selenoprotein T; *Selenov*, Selenoprotein V; *Selenow*, Selenoprotein W; *Sephs*2, Selenophosphate synthetase 2; *Txnrd*1, Thioredoxin reductase 1; *Txnrd*2, Thioredoxin reductase 2; *Txnrd*3, Thioredoxin reductase 3.

The kidney displayed unique expression profiles reflective of its specialized physiological functions (Figure 4 and Figure S4). *Dio* gene expression was either undetectable or minimal during embryogenesis (Figure 4A). *Dio*1 increased sharply (>70-fold, *p* < 0.05) between E18.5 and P7, then decreased approximately 30-fold by P90. *Dio*2 and *Dio*3 exhibited moderate increases (~6-fold) from E18.5 to P7 before declining. Similarly, all four *Gpx* genes were barely detectable during embryonic development (Figure 4B). *Gpx*1, *Gpx*3, and *Gpx*4 increased sharply from P7 to P30 (>150-fold, >100-fold, and >20-fold, respectively; *p* < 0.05) before decreasing, while *Gpx*2 remained low and stable throughout development.

As shown in Figure 4C, *Txnrd*2 and *Txnrd*3 increased sharply from E8.5, peaking at P30 (>150-fold and >50-fold, respectively; *p* < 0.05) before decreasing. *Txnrd*1 increased approximately 25-fold from E8.5 to E12.5 before declining continuously to baseline levels. *Selenoi* exhibited a slight but consistent increase between E12.5 and P90 (Figure 4D). *Msrb*1 increased steadily from E18.5 to a peak at P90. *Sephs*2 and *Selenop* demonstrated low or stable expression during early postnatal stages, with *Sephs*2 increasing sharply and linearly (>200-fold, *p* < 0.05) from P30 to P90 (Figure 4E), and *Selenop* increasing sharply and linearly (>500-fold, *p* < 0.05) from E18.5 to P90.

*Selenoh* maintained low and stable expression levels (Figure 4F). *Selenov* was undetectable in renal tissue. *Selenom* increased moderately (>4-fold, *p* < 0.05) from E18.5 to P7 before decreasing by half (Figure 4G). *Selenof*, *Selenoo*, *Selenot*, and *Selenow* exhibited low and stable expression during embryogenesis. *Selenoh* and *Selenot* increased sharply (~30-fold and ~18-fold, respectively; *p* < 0.05) between E18.5 and P7 before decreasing linearly to baseline levels. *Selenoo* increased moderately (~5-fold) from P7 to P30 before decreasing. *Selenow* remained low and stable throughout development. *Selenok* and *Selenos* showed low expression during embryogenesis but increased moderately (~4-fold, *p* < 0.05) from E18.5 to P7 before declining (Figure 4H). *Selenon* increased from E8.5 to a peak at E18.5, decreased approximately 20-fold by P7, peaked again at P30, and then decreased sharply.

## 4. Discussion

This study provides the first comprehensive developmental atlas of the murine selenoproteome, quantifying mRNA expression of all 24 selenoprotein genes across four vital organs at six critical developmental stages. Our data reveal highly dynamic, tissue-specific, and stage-dependent regulatory patterns, significantly advancing our understanding of selenoprotein roles in embryonic and postnatal development [26,27,28,29]. The findings offer important insights into the molecular mechanisms through which selenium—an essential human micronutrient—supports developmental processes and maintains tissue homeostasis. Below, we discuss our key findings in the context of current knowledge and their physiological implications, with particular attention to potential relevance for human nutrition and health.

### 4.1. Expression Dynamics Across Gene Families and Tissues

Our results demonstrate that developmental expression patterns are predominantly gene- and tissue-specific, with limited conservation across entire protein families. For instance, the glutathione peroxidase family (*Gpx*1-4) exhibited divergent regulation: *Gpx*1 and *Gpx*3 showed significant postnatal upregulation in multiple tissues, whereas *Gpx*2 and *Gpx*4 expression remained relatively stable throughout development. Similarly, the deiodinases (*Dio*1-3) were largely transcriptionally silent during embryogenesis but were significantly upregulated postnatally in all tissues examined. This absence of a uniform regulatory pattern underscores that developmental control of the selenoproteome is not monolithic but is precisely tailored to the functional requirements of specific genes within their tissue microenvironments.

The observed tissue-specific patterns likely reflect the specialized physiological functions of each organ. The pronounced postnatal expression of *Gpx*3 in the kidney aligns with its established role as a plasma glutathione peroxidase primarily produced by renal cells [30]. This finding has implications for human renal health, as GPX3 polymorphisms have been associated with cardiovascular and renal pathologies in epidemiological studies. Conversely, the dramatic induction of *Dio*1 and *Dio*2 in the liver postnatally corresponds to its central role in systemic thyroid hormone metabolism [25]. These organ-specific expression profiles suggest that selenoproteins have evolved to meet the unique redox and metabolic demands of different tissues during development [31], and understanding these patterns may inform targeted nutritional strategies for organ-specific selenium support in humans.

### 4.2. Expression Characteristics of Selenoproteins Essential for Embryonic Survival

A pivotal finding was that the five selenoprotein genes whose individual knockout results in embryonic lethality (*Gpx*4, *Txnrd*1, *Txnrd*2, *Selenoi*, *Selenot*) did not share a common high-abundance expression pattern during embryogenesis. Instead, most were expressed at low to moderate levels. This suggests that their essentiality for survival stems from their indispensable biological functions (e.g., *Gpx*4 in preventing lethal lipid peroxidation, *Txnrd*2 in supporting hematopoiesis) rather than their expression magnitude. Their knockout proves lethal not because they are highly expressed, but because they perform non-redundant, critical functions that cannot be compensated by other mechanisms, even at low expression levels [9,10,11,12].

The expression profile of *Gpx*4 is particularly instructive. Despite being essential for embryonic survival as early as E7.5, its expression remained relatively stable across development in all tissues examined. This indicates that even basal levels of *Gpx*4 are sufficient for its vital function in preventing lipid peroxidation and supporting embryonic development, but that its complete absence cannot be tolerated. Similarly, *Txnrd*1 and *Txnrd*2 showed only modest expression during embryogenesis despite their essential roles, reinforcing the concept of qualitative essentiality superseding quantitative abundance [30,32,33]. The expression of *Msrb*1, which repairs methionine sulfoxidation in proteins, was notably high in the liver and heart but relatively low in the brain despite the high mitochondrial density in the latter. This suggests that the brain may rely on alternative redox systems, such as the mitochondrial thioredoxin reductase *Txnrd*2, which showed high expression in the brain. Similarly, the limited induction of GPx enzymes in brain and heart, tissues with high aerobic metabolic rates, may be compensated by other antioxidant systems. Another striking finding was the general decline in selenoprotein expression from adolescence (P30) to adulthood (P90), except for *Msrb*1 which increased with age. This may reflect a reduced demand for selenium-dependent antioxidant defense after the rapid growth phase, or a shift to other maintenance mechanisms.

### 4.3. Developmental Regulation of Selenoprotein Biosynthesis Genes

The expression of genes central to selenoprotein biosynthesis, *Selenop* and *Sephs*2, was notably early and dynamic. *Selenop* expression was detectable as early as E8.5 in the liver, consistent with its role as the primary selenium transport protein. Its significant postnatal increase across tissues aligns with the heightened demand for selenium distribution to support rapid growth and selenoprotein synthesis in developing organs. The expression pattern of *Sephs*2, which synthesizes the selenium donor for Sec incorporation, was more variable but generally increased during development. This indicates that the molecular machinery for selenium utilization is established early in embryogenesis and is further amplified to meet the metabolic demands of postnatal life.

The coordinated expression of these biosynthesis genes suggests a sophisticated regulatory system ensuring adequate selenium supply and utilization during critical developmental windows. The early expression of *Selenop* is particularly crucial given that maternal-fetal selenium transfer occurs primarily through this protein [34]. This finding underscores the importance of maternal selenium status for fetal development and has direct implications for human prenatal nutrition recommendations. The postnatal increase in both *Selenop* and *Sephs*2 corresponds to the period of rapid growth and increased metabolic activity, where selenium demand is substantially heightened [30,31]. Understanding these developmental patterns can inform optimal timing for selenium supplementation in human infants and children.

### 4.4. Conservation and Divergence of Expression Across Tissues

Certain genes exhibited consistent expression patterns across most tissues. For example, *Gpx*4 maintained stable expression from embryogenesis through adulthood in all four organs, highlighting its fundamental role in universal cellular homeostasis. Conversely, other genes displayed striking tissue specificity. *Gpx*3 expression was uniquely and highly upregulated in the kidney postnatally, consistent with its known role as a plasma antioxidant often produced by renal cells. The thyroid hormone-activating enzymes *Dio*1 and *Dio*2 showed dramatic postnatal induction in the liver, a key organ for thyroid hormone metabolism, but not to the same extent in the heart or brain. These patterns reflect the specialized physiological functions of different tissues.

The brain exhibited particularly distinctive expression patterns, with several selenoproteins showing delayed or attenuated postnatal induction compared to other tissues. This may reflect the brain’s unique redox environment and specialized protective mechanisms. The complementary expression of *Selenop* and *Sephs*2 in the brain suggests an elaborate local regulatory mechanism to maintain selenium homeostasis, which is of paramount importance given the brain’s high susceptibility to oxidative stress [35]. This dependency is underscored by the high expression of a specific set of selenoproteins, particularly those resident in the endoplasmic reticulum (ER) such as SELENOK, SELENOS, SELENOM, and SELENOT, which are crucial for mitigating neuronal ER stress, regulating calcium flux, and ensuring quality control of synaptic proteins [36]. Consequently, the brain’s distinctive selenoprotein expression profile, as observed in our study, equips it with specialized protective mechanisms to safeguard its function despite its high metabolic demand and oxidative environment [35,36]. These findings highlight the particular importance of adequate selenium nutrition for human brain development and function, with potential implications for preventing neurodegenerative disorders.

### 4.5. Implications for Tissue-Specific Vulnerability to Selenium Deficiency

The collective expression patterns within a tissue likely predispose it to specific pathological phenotypes upon selenium deficiency. For example, the heart and skeletal muscle (not directly studied here but relevant) express high levels of *Selenon*. The well-documented sensitivity of heart and skeletal muscle to selenium deficiency, manifesting as nutritional myodegeneration, has been linked to selenoproteins including *Selenon*. Similarly, the brain’s unique expression pattern, including the coordinated expression of *Selenop* and *Sephs*2, may represent an adaptive mechanism to maintain selenium homeostasis and protect against oxidative stress in this vulnerable organ, explaining the neurological sequelae observed in deficiency states [31].

The liver’s expression profile, characterized by high levels of multiple selenoproteins including *Selenop*, *Gpx*1, and *Dio*1, underscores its central role in whole-body selenium metabolism and its relative resistance to selenium deficiency compared to other tissues. The kidney’s unique pattern, with high expression of *Gpx*3 and several other selenoproteins, aligns with its role in selenium reabsorption and redistribution. This tissue-specific expression pattern may explain why certain organs, such as the heart, are particularly vulnerable to selenium deficiency in humans, as evidenced by the occurrence of Keshan disease—a selenium-responsive cardiomyopathy—in selenium-deficient regions [32]. Understanding these tissue-specific vulnerabilities can guide targeted nutritional interventions for populations at risk of selenium deficiency.

### 4.6. Metabolic Transitions and Corresponding Expression Changes

The observed mRNA expression patterns are closely correlated with major metabolic transitions during development. The embryonic environment is relatively hypoxic and relies heavily on glycolytic energy production. The transition to postnatal life introduces a hyperoxic environment and a shift towards oxidative metabolism for energy production, generating substantially higher levels of reactive oxygen species (ROS). The significant postnatal upregulation of many antioxidant selenoproteins like *Gpx*1, *Gpx*3, and *Txnrd*3 represents a crucial adaptation to this new metabolic reality, providing enhanced capacity to mitigate oxidative damage. Furthermore, the postnatal surge in *Dio*1 and *Dio*2 expression aligns with the increased need to activate thyroid hormone, a master regulator of thermogenesis and metabolic maturation after birth [33,37].

The timing of these expression changes suggests that selenoproteins are precisely regulated to meet the changing metabolic demands of development. The early expression of certain selenoproteins during embryogenesis may protect against developmental oxidative stress, while the postnatal induction of others supports the transition to extrauterine life and the increased metabolic activity of mature tissues. These developmental metabolic transitions have direct parallels in human development, suggesting that selenium requirements may vary across life stages.

### 4.7. Limitations and Future Perspectives

A primary limitation of this study is its exclusive focus on mRNA abundance, which may not always directly correlate with protein levels or enzymatic activity due to post-transcriptional regulatory mechanisms. This is particularly relevant for selenoproteins, whose synthesis involves complex translational machinery and is highly dependent on selenium availability. Furthermore, the biological rationale underlying the expression pattern of some genes, such as the consistently low embryonic expression of crucial genes like *Txnrd1/2*, remains an intriguing area for future investigation. It suggests exquisite precision in redox management during early development. The absence of *Selenov* mRNA in all tested somatic tissues was confirmed, reinforcing its testis-specific expression pattern. In addition, when collecting tissues for qPCR measurement of selenoprotein expression, cellular heterogeneity may affect the expression profile, as the technique does not account for changes in cell-type composition during development. Future studies using single-cell RNA sequencing could elucidate these cell-type-specific expression patterns. Future studies should integrate proteomic approaches to quantify actual protein levels and enzymatic activities across development. Functional investigations using tissue-specific and inducible knockout models would help elucidate the specific roles of individual selenoproteins in different organs during critical developmental windows. Additionally, exploring the regulatory mechanisms controlling selenoprotein expression, including transcription factors, epigenetic modifications, and the influence of selenium status, would provide deeper insights into the developmental regulation of the selenoproteome. Translational studies examining how dietary selenium forms and levels influence these developmental expression patterns could provide valuable insights for human nutrition.

## 5. Conclusions

This study provides an unprecedented atlas of selenoprotein gene expression throughout murine development, offering insights with potential relevance to human nutritional science. We demonstrate that the selenoproteome is under precise spatiotemporal control, reflecting the specific functional demands of developing tissues. The expression patterns of genetically essential genes highlight the principle of qualitative essentiality overriding quantitative abundance, while the dynamics of biosynthesis and antioxidant genes mirror fundamental developmental metabolic transitions. These findings significantly advance our understanding of selenium biology in development and provide a robust framework for investigating the molecular mechanisms underlying the pathologies of selenium deficiency. Furthermore, our results suggest that optimal selenium nutrition during critical developmental windows may be essential for supporting proper organ development and function, with implications for human maternal and child health nutrition strategies.

## Figures and Tables

**Table 1:** Oligonucleotide sequences of selenoprotein genes in mice.

Genes	Oligonucleotide (5′–3′)	Oligonucleotide (5′–3′)
*β-actin*	CTATTGGCAACGAGCGGT	GGTCTTTACGGATGTCAACG
*GAPDH*	GGTGCTAAGCGTGTTATCATCTCA	CATGGTTGACACCCATCACAA
*Dio1*	GGAACCATAGGCATTGGAAA	AGTGCCAGAGAGCCAGATTC
*Dio2*	GTGCTGATGTGTTGTTCCTGCCAA	TACACCCTTCACTCAGCACCCAAA
*Dio3*	AGCGCAGCGAGAGTACTACAACAA	ACATGATGGTGCCACTCTGGATGA
*Gpx1*	GGTTCGAGCCCAATTTTACA	CCCACCAGGAACTTCTCAAA
*Gpx2*	GCATGGCTTACATTGCCAAGTCGT	AGCCCTGCCTCTGAACGTATTGAA
*Gpx3*	GATGTGAACGGGGAGAAAGA	CCCACCAGGAACTTCTCAAA
*Gpx4*	CTCCATGCACGAATTCTCAG	ACGTCAGTTTTGCCTCATTG
*Msrb1*	ACAGTTGTTGCCCCATTAGC	GGAGTGGGTCTCAGCTTCAG
*Selenof*	CTCACCAGTGAAACGCTTTG	TCAAAGAGCACACAGCAAGG
*Selenoh*	GCGAGATTTGAACTTTGCATC	TTGTCCACCGTCTCCATAGG
*Selenoi*	ATAGTGACTGCGGTTGTGGGAGTT	TGGCTTCATAGACGGACTTGTGCT
*Selenok*	GCTGGTGGATGAGGAAGGTA	CTCATTCATCTGTGGGGACA
*Selenom*	TTTGTCACCGAGGACATTCA	TGTACCAGCGCATTGATCTC
*Selenon*	TGACTGCCATCAGCGATTCCTACT	GTGATTGGGCACAAACAGCCTGAA
*Selenoo*	TGCACAGAAAGCCATTGAAG	GGAAGATTGCTCCTCAGTGC
*Selenop*	GCAATTGCTTGACAGTGTGC	TTCATGGGCTGATTTTGTCA
*Selenos*	GCCTTACGCACACTTTCACA	GTGGCCTAATGGCAATGTCT
*Selenot*	TGTGGCAACAGAAAGGGATT	CAGGTGGCATCAACATCAAG
*Selenov*	ACCCTGGAGCATCAATTCCCAAAC	ACCCACAAGTTTCTTCAGACCGGA
*Selenow*	CCCAAGTACCTCCAGCTCAA	GCCATCACCTCTCTTCTTGG
*Sephs2*	TGCCAATGTGCTCAGTGACCTCTA	TTCACTCATGCTCTGGCTCACACT
*Txnrd1*	TTCGACCTGATCATCATTGG	CCACATTCACACACGTTCCT
*Txnrd2*	CCCTAGAGTGTGCTGGCTTC	AAGCATGATCCTCCCAAGTG
*Txnrd3*	CCTTTCCCAGTTGCTAGTGC	GTGCTACACTCTGGGCAACA

## Data Availability

Data will be made available on request.

## Supplementary Materials

The following supporting information can be downloaded at https://www.mdpi.com/article/10.3390/nu17203200/s1, Figure S1: Heatmap of selenoprotein genes expression in heart of mice during embryo and postnatal stages. Figure S2: Heatmap of selenoprotein genes expression in brain of mice during embryo and postnatal stages. Figure S3: Heatmap of selenoprotein genes expression in liver of mice during embryo and postnatal stages. Figure S4. Heatmap of selenoprotein genes expression in kidney of mice during embryo and postnatal stages.

## Abbreviations

SEM, Standard Error of the Mean; *ActB*, *β-actin*; *gapdh*, glyceraldehyde-3-phosphate dehydrogenase; cDNA, Complementary DNA; CXXU, Cys-x-x-Sec; PCR, polymerase chain reaction; mRNA, messenger RNA; Q-PCR, real-time quantitative polymerase chain reaction; Se, selenium; Sec, selenocystein; *Dio1*, Iodothyronine deiodinases 1; *Dio2*, Iodothyronine deiodinases 2; *Dio3*, Iodothyronine deiodinases 3; *Gpx1*, glutathione peroxidase 1; *Gpx2*, glutathione peroxidase 2; *Gpx3*, glutathione peroxidase 3; *Gpx4*, glutathione peroxidase 4; *Msrb1*, Selenoprotein R; *Selenof*, Selenoprotein F; *Selenoh*, Selenoprotein H; *Selenoi*, Selenoprotein I; *Selenok*, Selenoprotein K; *Selenom*, Selenoprotein M; *Selenon*, Selenoprotein N; *Selenoo*, Selenoprotein O; *Selenop*, Selenoprotein P; *Selenos*, Selenoprotein S; *Selenot*, Selenoprotein T; *Selenov*, Selenoprotein V; *Selenow*, Selenoprotein W; *Sephs2*, Selenophosphate synthetase 2; *Txnrd1*, Thioredoxin reductase 1; *Txnrd2*, Thioredoxin reductase 2; *Txnrd3*, Thioredoxin reductase 3.
