# Supplementary material for: Developmental Regulation of the Murine Selenoproteome Across Embryonic and Postnatal Stages: Implications for Human Nutrition and Health
Source: Nutrients. 2025 Oct 11;17(20):3200. doi: 10.3390/nu17203200 (PMC12566671; doi:10.3390/nu17203200)
Supplement: Supplementary file 1 [file nutrients-17-03200-s001.zip › nutrients-3882863-supplementary.pdf]

Supporting Information

Developmental Regulation of the Murine Selenoproteome Across Embryonic and Postnatal Stages: Implications for Human Nutrition and Health

Shan-Shan Wang [1], Tong Li [3], Cheng-Jia Wei [2] and Lan-Yu Cui [2,*]

[1] College of Food Science and Nutritional Engineering, China Agricultural University, Beijing 100083, China; 18064636815@163.com
[2] University Engineering Research Center of Advanced Technologies in Medical and Biological Intelligent Manufacturing, Guangxi Medical University, Key Laboratory of Longevity and Aging-Related Diseases of Chinese Ministry of Education, Institute of Neuroscience, School of Basic Medical Sciences, Guangxi Key Laboratory of Brain Science, Key Laboratory of Basic Research on Brain Function and Disease of Guangxi Health Commission, Nanning 530021, China; myqfchenguang@163.com
[3] Institute of Agricultural Products Preservation and Processing Technology (National Engineering and Technology Research Center for Preservation of Agricultural Products), Tianjin Academy of Agricultural Sciences, Key Laboratory of Storage and Preservation of Agricultural Products, Ministry of Agriculture and Rural Affairs, Tianjin 300384, China
* Correspondence: cuily12@tsinghua.org.cn

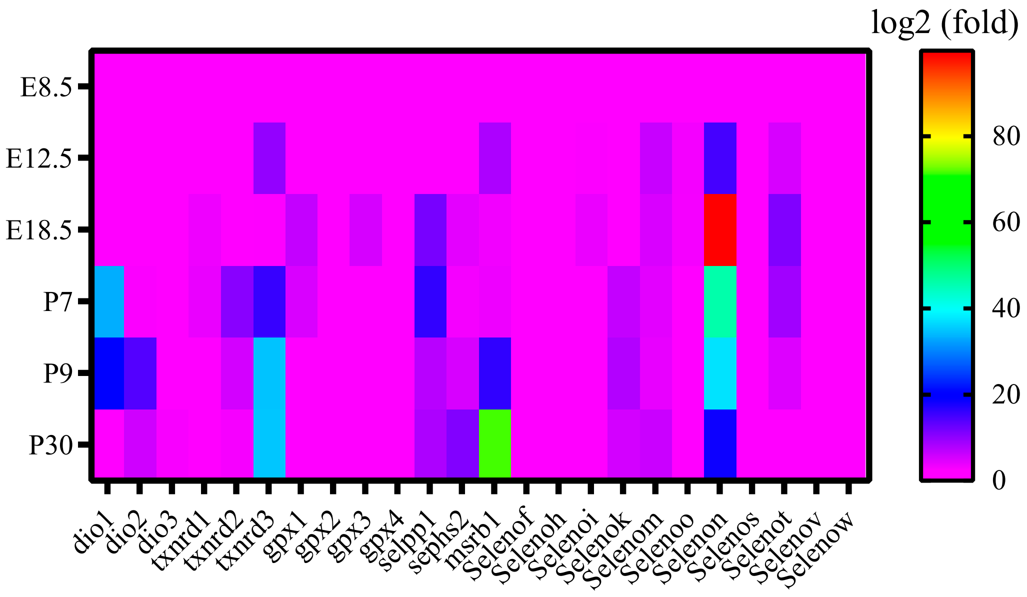

Figure S1. Heatmap of selenoprotein genes expression in heart of mice during embryo and postnatal stages.

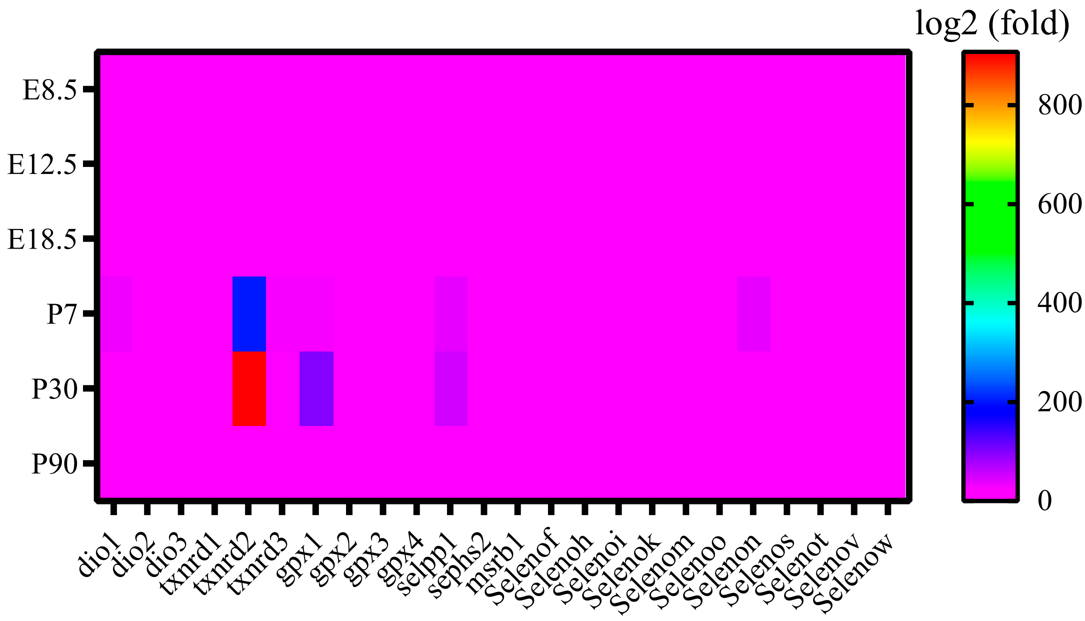

Figure S2. Heatmap of selenoprotein genes expression in brain of mice during embryo and postnatal stages.

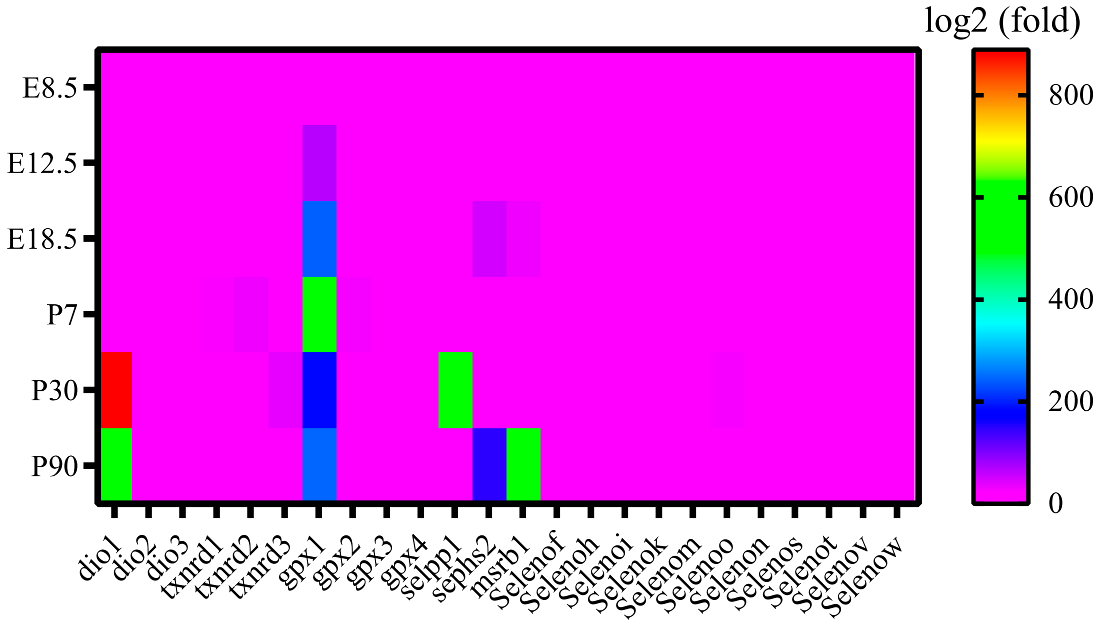

Figure S3. Heatmap of selenoprotein genes expression in liver of mice during embryo and postnatal stages.

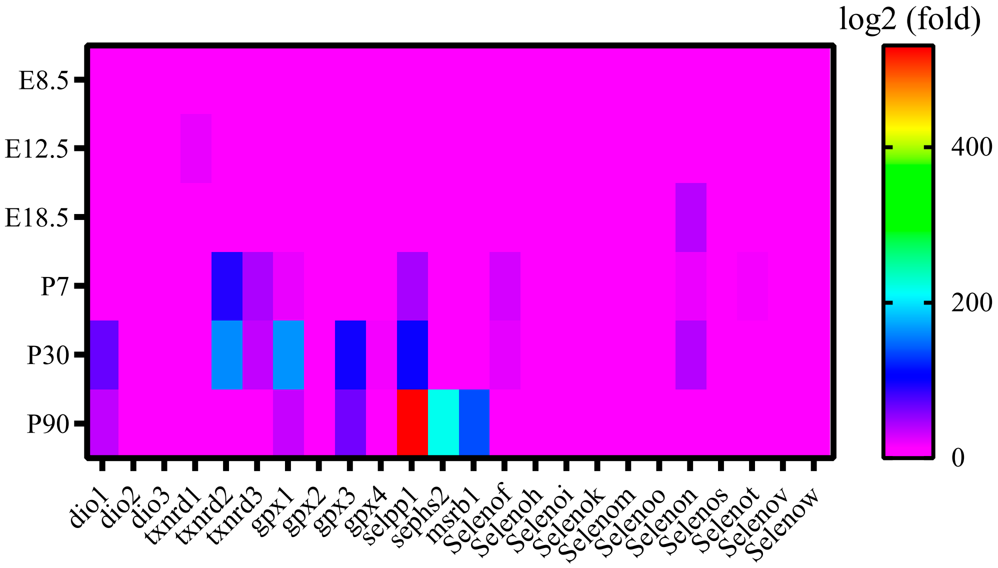

Figure S4. Heatmap of selenoprotein genes expression in kidney of mice during embryo and postnatal stages.
